# Inter-State Association of Livestock Sanitary Boards

**Published:** 1899-12

**Authors:** R. Johnson

**Affiliations:** President


### SOCIETY PROCEEDINGS.

### INTER-STATE ASSOCIATION OF LIVESTOCK SANITARY BOARDS.

THE third annual meeting was held at the Exchange Building, Union Stockyards, Chicago, on October 11 and 12, 1899. Sixteen States were represented by about forty-five delegates. Most of the States that participated in the meeting were Western and Central Western States. The only Eastern States represented were Pennsylvania and Massachusetts. This organization held its last meeting in Omaha, and the one before that in Fort Worth, Texas. It has always devoted its energies largely to the matter of quarantining and protecting the North against cattle that may carry Texas cattle-ticks. The membership is composed of the State officers charged with the control of the diseases of animals, and most of these are members of the State Livestock Sanitary Boards.

The first address, Wednesday morning, was by Dr. D. E. Salmon, on the subject of Texas fever. Dr. Salmon reported on the dipping experiments that have been carried on by the government for several years, in which attempts have been made to discover a means of dipping Southern cattle that will not injure the cattle but will destroy the Southern cattle-tick, the carrier of the germs of Texas fever. If this could be accomplished it would make it possible for the government to relax the present quarantine on Southern cattle, permitting them to come North at any time and for any purpose after they have been properly dipped and inspected. Unfortunately, no means of destroying the tick has yet been found that is not injurious to the cattle. Various solutions and compositions that have been tried will injure cattle if strong enough to kill the tick. There are many cattle in the South that are ready for shipment, and many stockmen in the North who would like to buy such cattle if they could do so safely. A committee on quarantine line in open season, composed of one member from each State represented, reported in favor of the present quarantine line, with the exception that three counties in Tennessee that are now below the line should be placed above it. These counties are Cannon, Warren, and Lincoln. It is also agreed to recommend to the Secretary of Agriculture that the open season this year shall extend from November 1st to January 1st. The representatives from Kansas were somewhat opposed to an open season, as they said that Texas fever had occurred in Kansas following the importation of Texas cattle in the month of December.

The subjects of sheep-scab and tuberculosis were freely discussed. Resolutions were adopted indorsing the position that the Department of Agriculture has taken in relation to the dipping of sheep.

The following report of the Committee on Line and Open Season was presented by the Secretary and was unanimously adopted:

*Resolved,* That the Interstate Association of Livestock Sanitary Boards respectfully recommends to the Secretary of Agriculture the establishment for the ensuing year of the present quarantine line bounding the district to be scheduled, on account of danger from Southern cattle fever, with the exception that Cumberland, Cannon, and Lincoln Counties, in Tennessee, be placed above the line, upon the State sanitary authorities of that State furnishing to the Department satisfactory proof that said counties are free from infestation with *Boophilis bovis.*

*Resolved*, That this Association recommends the withdrawal of the quarantine regulations applying to cattle on account of Texas fever infection from November 1st to January 1st; provided that all cattle from below the quarantine line destined for feeding in the States of Missouri, Kansas, and Texas, and the Territories of Oklahoma, New Mexico, and Arizona, be inspected and allowed only to proceed in case they are found free from ticks; and provided, further, that all infected cattle in transit through the States and Territories mentioned be treated as are infected cattle during the quarantine season.

WHEREAS, There is no doubt that on November 1st, when the open season under the present quarantine regulations will commence, there are in the southern divisions of all Northern stockyards numerous young living ticks (*Boophilis bovis*), which would render it dangerous for native cattle to be yarded in said southern divisions prior to the occurrence of sufficient cold to destroy such ticks; therefore

*Resolved*, That this Association respectfully petitions the Secretary of Agriculture to issue orders prohibiting the yarding of any native cattle in the southern division of stockyards north of the quarantine line at any season of the year.

The following resolutions reported by the Committee on Resolutions were unanimously adopted:

WHEREAS, Tuberculosis of cattle is now attracting much attention in all parts of the country, and has been the subject of much legislation in the Eastern States and in some of the Central States; and

WHEREAS, In some of the discussions on this subject certain fundamental facts have been lost sight of or are misstated, and there is need of a definite statement as to some of the points in regard to tuberculosis that are so fully established as to be placed beyond reasonable doubt, and have been accepted and indorsed by the conventions that have taken action on this subject during recent years; be it

*Resolved*, That the following points in regard to tuberculosis may be considered established, and should enter into all plans for controlling this disease:

1. Tuberculosis is a contagious disease.
2. Tuberculosis prevails extensively in many parts of the United States, and is spreading, excepting in unstabled beef-producing herds and in States that are combating it actively.
3. While the losses caused among cattle by this disease are large and sufficient to justify energetic efforts to eradicate it, tuberculosis is also of much importance on account of the intimate relation of this disease of cattle to the public health.
4. Tuberculin furnishes by far the most accurate means that is at present available for recognizing tuberculosis in living animals, and the mistakes made where it is carefully used are so few as to be of no practical importance.
5. It is necessary that States shall authorize measures planned to suppress this disease if it is not to be permitted to continue to spread.

6. This convention recommends that all States that have not already done so shall authorize and empower the proper authorities to adopt well-planned and conservative measures to repel the encroachments of this disease and to eradicate it where it is already established.

WHEREAS, The contagion of sheep-scab has been and is now being widely disseminated through channels of interstate commerce; and

WHEREAS, Said disease may be easily and cheaply cured by proper dipping; and

WHEREAS, The Secretary of Agriculture has recently promulgated regulations requiring the dipping of diseased sheep found in transit from one State to another; therefore be it

*Resolved*, That this convention expresses its appreciation of the efforts of the Department of Agriculture to repress and control sheep-scab, and recommends the rigid enforcement of such measures as may be considered necessary to secure this result.

R. JOHNSON,
President.

